# Infrared Predictions Are a Valuable Alternative to Actual Measures of Dry-Cured Ham Weight Loss in the Training of Genome-Enabled Prediction Models

**DOI:** 10.3390/ani12070814

**Published:** 2022-03-23

**Authors:** Valentina Bonfatti, Sara Faggion, Elena Boschi, Paolo Carnier

**Affiliations:** Department of Comparative Biomedicine and Food Science, University of Padova, Viale dell’Università 16, 35020 Legnaro, Italy; sara.faggion@unipd.it (S.F.); elena.boschi@phd.unipd.it (E.B.); paolo.carnier@unipd.it (P.C.)

**Keywords:** ham quality, infrared spectroscopy, animal breeding, genomic selection, pig

## Abstract

**Simple Summary:**

Excessive ham weight losses during dry-curing (WL) result in a loss of marketable product, hindering the quality of dry-cured hams. Genetic selection for reducing WL requires individual traceability of hams throughout the dry-curing process and the measurement of WL is expensive, time-consuming, and can be performed only at the end of seasoning, resulting in long generation intervals. Infrared spectroscopy provides early, cost-effective, high-throughput predictions of WL that are highly genetically correlated with the actual measures. This study focused on the accuracy of genomic prediction models for observed and infrared-predicted WL. Models were tested on crossbred pigs and their purebred sires in random cross-validation and in a leave-one-family-out training-validation scheme. Accuracy of prediction of sire genetic merit, estimated from crossbred training data for actual ham WL, was 0.38, slightly higher than the accuracy attainable by a model trained on infrared-predicted WL (0.32). While the accuracy of genomic predictions is satisfactory for both the observed and infrared-predicted WL, the use of infrared predictions results in considerably lower phenotyping costs, enabling the construction of larger reference populations.

**Abstract:**

Selection to reduce ham weight losses during dry-curing (WL) requires individual traceability of hams throughout dry-curing, with high phenotyping costs and long generation intervals. Infrared spectroscopy enables cost-effective, high-throughput phenotyping for WL 24 h after slaughter. Direct genomic values (DGV) of crossbred pigs and their purebred sires were estimated, for observed (OB) and infrared-predicted WL (IR), through models developed from 640 and 956 crossbred pigs, respectively. Five Bayesian models and two pseudo-phenotypes (estimated breeding value, EBV, and adjusted phenotype) were tested in random cross-validation and leave-one-family-out validation. The use of EBV as pseudo-phenotypes resulted in the highest accuracies. Accuracies in leave-one-family-out validation were much lower than those obtained in random cross-validation but still satisfactory and very similar for both traits. For sires in the leave-one-family-out validation scenario, the correlation between the DGV for IR and EBV for OB was slightly lower (0.32) than the correlation between the DGV for OB and EBV for OB (0.38). While genomic prediction of OB and IR can be equally suggested to be incorporated in future selection programs aiming at reducing WL, the use of IR enables an early, cost-effective phenotyping, favoring the construction of larger reference populations, with accuracies comparable to those achievable using OB phenotype.

## 1. Introduction

While dehydration during ham dry-curing is essential for hindering the onset of anomalous fermentations and ensures the development of the typical sensory attributes, excessive ham weight losses (WL) lead to a loss of marketable product and impair the quality of the dry-cured ham [1]. For this reason, WL is one of the traditional breeding goals of the Italian pig breeds (Large White, Landrace, and Duroc) used to produce Italian protected designation of origin (PDO) hams. Currently, according to [2], the breeding program of any breed and line used for the production of Italian PDO hams must comply with two requirements: maintain or increase pig subcutaneous fat thickness, and maintain or improve the aptitude of the meat to seasoning. Hence, including WL among the breeding goals of the genetic types intended for PDO ham production has implicitly become mandatory.

Even though WL is moderately heritable [3,4], genetic selection aiming at reducing WL is challenging because (1) measures of WL require the individual traceability of hams throughout the dry-curing process, largely limiting the availability of phenotypes, and (2) phenotyping for WL requires completion of the entire dry-curing process (which lasts at least 400 days, according to the most recent revision of the San Daniele ham specification [5]), resulting in long generation intervals and reduced response to selection. To overcome these limitations, since the early nineties, the Italian pig breeder association (ANAS, Rome, Italy) implemented an indirect selection for WL in the Large White, Landrace, and Duroc breeds, based on the ham weight loss after 7 d of salting [6]. This strategy, although successful in the long term, exploits a moderate additive genetic correlation (~0.65) between the ham weight loss after 7 d of salting and WL [6] and requires the availability of facilities for ham processing that can guarantee the traceability of individual hams.

Infrared spectroscopy is the method of choice to enable rapid and non-destructive prediction of green ham subcutaneous fat quality and monitor its compliance with the requirements on fat composition dictated by PDO ham specifications [5,7]. This technology, besides being a potential phenotyping tool for fat quality [8], has also been successfully used to predict different technological and sensory attributes of dry-cured hams [9,10]. Recently, on-site infrared spectroscopy has demonstrated its effectiveness as a large-scale phenotyping tool for WL, by enabling early, cost-effective and high-throughput predictions of WL, based on spectra acquired on hams 24 h after slaughter combined with carcass and green ham quality traits [3]. The additive genetic correlation between the observed WL (OB) and its infrared prediction (IR) is positive and high (~0.88) [3]. Such correlation and the higher heritability of IR compared to OB [3] suggest the incorporation of IR in selective breeding programs to overcome limitations of WL phenotyping. 

The adoption of genomic selection might increase the effectiveness of selective breeding practices aimed at decreasing WL [11,12]. In pig populations, the accuracy of genome-enabled prediction of breeding values declines rapidly over generations, and a periodical collection of phenotypes is needed to retrain and update genomic prediction models [13,14,15]. Incorporation of IR into genomic selection programs for WL in place of OB would facilitate the periodical phenotype collection at virtually no additional costs and the creation of larger reference populations for training and validation of genomic models. However, the feasibility of developing genomic selection strategies based on IR depends on the accuracy of selection. The accuracy of genomic predictions for WL has never been investigated before, and no reports are available on the comparison between the predictive ability of genomic models trained on actual observations of a trait or on its infrared predictions in pigs. Hence, the objectives of this study were to assess the accuracy of genome-enabled predictions of OB and IR and to evaluate, relative to models trained on OB, the ability of genomic models trained on IR to predict phenotypes or breeding values for WL.

## 2. Materials and Methods

### 2.1. Phenotypes

Phenotypes were collected in a population of crossbred finishing pigs (CB) generated by the nucleus boars of the C21 Goland sire line (Gorzagri, Fonzaso, Italy) in the sib-testing program of the line. Since 2001, the breeding goal of the sire line includes traits related to the quality of dry-cured hams [4]. Details on the sib-testing program, parental lines, and farming conditions were described in [4]. Pigs were all raised on the same fam and slaughtered in the same commercial slaughterhouse under standardized conditions in batches of about 70 animals each. Age at slaughter was ≥270 days, and body weight was ≥160 kg (on average 167 ± 15 kg), in compliance with the main Italian PDO dry-cured ham specifications [5,7].

A measure of WL at the end of dry-curing (368 ± 4 days) was obtained for the left ham of 1888 CB slaughtered between December 2015 and July 2017. Of these pigs, 1624 had also a record of visible-infrared spectra acquired from the green ham subcutaneous fat and were used to develop near-infrared prediction models for WL. Infrared predictions of WL were obtained by a parametric regression model, namely Bayes B, implemented in the BGLR package [16] of the R software [17], as described in detail by [3]. Briefly, the prediction model included the following explanatory variables: (1) on-site visible-infrared spectra, acquired from the transversal section of subcutaneous fat of the green ham with a LabSpec^®^5000 (ASD Inc., Boulder, CO, USA) working over the spectral range between 350 and 2500 nm, and equipped with a fibre-optic contact probe; (2) sex; (3) carcass traits (weight, backfat depth and lean meat content of the carcass and total weight of the green hams); (4) green ham quality traits (subcutaneous fat depth and linear scores for round shape, subcutaneous fat thickness and marbling).

To obtain the IR for the 1624 CB with available data on OB, a set of prediction models was developed by excluding all the pigs of a given slaughter batch from the training set used to obtain model solutions and by applying the model solutions to calculate the IR for the batch that was left out. The process was repeated until the IR was obtained for the pigs of all slaughter batches (n = 25). Such a procedure mimics the scenario under which models are applied in practice, where predictions for new slaughter batches come from models trained on data of previous batches. It also minimizes the risk of overestimating the accuracy of IR and of the corresponding genomic prediction accuracy. While in a random cross-validation setting (where animals from the same batch can be assigned to both the training and testing set) the correlation between IR and OB was 0.79 [3], under these more restrictive circumstances the correlation between IR and OB was 0.65. Finally, a model trained on the entire dataset (n = 1624) was applied to a large dataset extracted from the historical database of the sib-testing program of the Goland C21 sire line to obtain, retrospectively, IR for all animals with no record on OB. The dataset included records of carcass traits, green ham quality traits, and spectral variables for 8048 CB. Hence, a total of 9672 records of IR were available for this study.

### 2.2. Pseudo-Phenotypes Used for Genomic Predictions

Genomic prediction models were investigated following a multiple-step genomic evaluation approach, where pseudo-phenotypes considered for the estimation of genomic prediction equations were phenotypes pre-corrected for fixed effects (y_adj_) or estimated breeding values (EBV). Estimates of fixed effects and EBV for OB and IR were obtained by solving univariate animal models using REMLF90 of the BLUPF90 family of programs [18]. Complete pedigree information, since the foundation of the C21 line, was available for all CB with phenotypic records and their purebred sires (PB), whereas only the parents and grandparents were known for the dams of the CB. Sires and dams of the CB, being animals of different pig lines, were unrelated. The univariate animal model was as follows:

(1) y=Xb+Za+e,

where **y** was a vector of phenotypes for OB or IR; **b** was a vector of unknown non-genetic (fixed) effects which included sex (female and castrated male) and slaughter batch effects; **a** was a random vector of unknown animal additive genetic effects assumed to follow a normal probability density **a** ~ N(0, **A**$\sigma_{a}^{2}$), where **A** and $\sigma_{a}^{2}$ denote the pedigree-derived relationship matrix and the additive genetic variance, respectively; **X** and **Z** were incidence matrices relating **b** and **a**, respectively, to **y**; **e** was a vector of random residuals with **e** ~ N(0, **I**$\sigma_{e}^{2}$), where **I** and $\sigma_{e}^{2}$ denote an identity matrix of appropriate order and the residual variance, respectively. 

The pre-corrected phenotypes for OB and IR were obtained as follows:

(2) $$y_{\mathrm{adj}}=y-X\hat{b}$$

where $\hat{b}$ are model solutions for **b** of the univariate animal Model (1). Accuracies of EBV were computed as:

(3) $$r_{\mathrm{BV},\mathrm{EBV}}=\sqrt{1-\mathrm{PEV}/\sigma_{g}^{2}}$$

where PEV is the prediction error variance and $\sigma_{g}^{2}$ is the estimated additive genetic variance of the trait.

### 2.3. Genotyping and Genomic Quality Control

High-density genotypes (GeneSeek GGP Porcine HD 50 K array, Neogen Co., Lansing, MI, USA) were available for all PB. Genotypes at 8826 SNP, obtained using the GeneSeek GGP Porcine LD 9 K array (Neogen Co., Lansing, MI, USA) according to the manufacturer’s protocol, were available for 1029 CB, spanning four generations. Of these pigs, 640 had a record on OB and were offspring of 57 PB, and 956 had a record on IR and were offspring of 104 PB. Sire families with records on both OB and IR were 53. 

Genotypes of CB were subsequently imputed to the GeneSeek GGP Porcine HD 50 K array using Fimpute v. 2.2 [19]. All genotypes were subjected to quality control using PLINK v. 1.9 software (http://www.cog-genomics.org/plink2 (accessed on 22 March 2022)). SNP located on sex chromosomes, exhibiting call rate < 0.9 or minor allele frequency < 5% were discarded. After editing, a total of 29,559 SNPs were retained for the development of genomic prediction models. No DNA samples with call rate < 0.9 or animals with parent-progeny genotype conflicts were detected. Hence, all the genotyped animals were available for the analyses.

### 2.4. Development of Genomic Prediction Models

Imputed genotypes at the 29,559 SNPs were used as predictors of the pseudo-phenotypes. Five Bayesian regression models were fitted to the data using the BGLR package [16] in the R software [17]: Bayesian ridge regression, Bayes A, Bayes B, Bayes C, and Bayesian Lasso. The general model for genomic prediction expressed in matrix notation was:

(4) y=1μ+Wg+e

where **y** is a vector of pseudo-phenotypes (y_adj_ or EBV), *μ* is the model intercept, **g** is a vector of unknown marker allele substitution effects, **W** is a matrix of observed marker genotypes (coded as the number of copies of the B allele as defined in the Illumina genotyping system nomenclature) for each individual and each marker, and **e** is a vector of random residual effects.

The prior distribution assigned to **g** differed depending on the model, with values of the hyper-parameters corresponding to BGLR default settings [20]. A total of 500,000 Gibbs samples were generated to estimate the parameters of the models, with a burn-in period of 100,000 samples, and a thinning of 100 samples. When the EBV was used as a pseudo-phenotype, EBV accuracies that were computed in the first step using Equation (3) were used as weighting factors [21]. For all models, the predicted direct genomic values (DGV) were computed using the following equation:

(5) $${\mathrm{DGV}}_{i}=\overset{p}{\underset{j=1}{\sum }}w_{ij}{\hat{g}}_{j}$$

where *p* is the number of SNPs; *w_ij_* is the genotype, coded as for Model (4), of animal *i* at SNP *j*; and *ĝ_j_* is the allele substitution effect for SNP *j* that was estimated by solving Model (4). The estimated allele substitution effects were used to calculate the DGV of the CB in the testing set and those of their PB.

### 2.5. Model Training and Validation Scenarios

As data for OB and IR were available for CB only, all models were trained on CB pseudo-phenotypes. Performance of models in prediction was then assessed using data either from CB or PB. In validation, CB were used primarily to investigate the sensitivity of model accuracy to different validation schemes, as well as the accuracy attainable with different pseudo-phenotypes. As the focus individuals in a breeding program addressed to the enhancement of WL are the purebred candidates, the predictive performance of models was evaluated also for the PB to mimic the circumstances under which models are applied in practice. This gave rise to nine different training-validation scenarios which are summarized in Table 1. While scenarios 1 and 2 aimed at comparing the accuracy attainable by models trained on different OB pseudo-phenotypes, scenario 3 was used to mimic a more realistic situation, where a reference population of CB is used to provide prediction equations of the genetic merit of PB for OB. Scenarios 4 to 6 investigated the ability of models trained on IR pseudo-phenotypes to predict y_adj_ or EBV for IR. In a real genomic selection program for WL, IR would be a proxy variable for OB. Hence, the performance of models trained on IR and predicting pseudo-phenotypes for OB was assessed in scenarios 7 to 9. The accuracy estimated in these scenarios can be used to compare the efficiency of genomic selection based on IR with the one of genomic-aided selective breeding targeting OB directly. In particular, scenario 9 represents the reference for the application of models in practice.

### 2.6. Random Cross-Validation

Each of the 9 scenarios investigated was initially considered in a 5-fold random cross-validation procedure. Data on CB (n = 640 for OB, n = 956 for IR) were randomly split into five data segments of equal size. In each fold, four data segments (training set) were used to train the Bayesian models and to obtain solutions for allele substitution effects, whereas the remaining segment served as a validation set in which pseudo-phenotypes were predicted, according to (5), using solutions of models resulting from the analysis of the training set. The validation set was made of the CB not included in the training set (for scenarios 1, 2, 4, 5, 7, and 8) or PB (for scenarios 3, 6, and 9).

### 2.7. Leave-One-Family-Out Validation

Despite being the most used method for evaluating the predictive ability of models, random cross-validations can lead to optimistic estimates of model accuracy, as the training set may include close relatives of the individuals of the validation set. This is not an ordinary condition in a real genomic selection program. In those programs, selection is based on the DGV predicted from allele substitution effects estimated from data of a reference population not including full-sibs or offspring of the breeding candidates. For this reason, the prediction performance of models was also assessed in a leave-one-family-out validation procedure (LOFO). This approach mimics, as far as possible, the circumstances under which models are applied in practice. In the LOFO, a Bayesian Ridge Regression model was trained on pseudo-phenotypes for OB or IR excluding at each round the members of a sire family from the training set. The estimated allele substitution effects obtained at each round were then used to calculate the DGV of the PB and of each CB of the family that was excluded from the training set. 

The EBV used as pseudo-phenotypes in the LOFO were obtained by a bivariate analysis using Model (1) for both traits. The bivariate analysis included 1888 records for OB and 9672 records for IR. The use of the bivariate analysis increased the number of sire families for which EBV of both traits were available (n = 104). In this case, pigs included in the calculation of genomic model accuracy comprised also genotyped CB with no phenotypic record on OB. The number of CB with EBV available to compute model accuracy was 735 and 995 for OB and IR, respectively.

### 2.8. Evaluation of the Predictive Performance of Models

Correlations between pseudo-phenotypes (y_adj_ or EBV) and DGV for animals in the validation sets were used as measures of model accuracy and to evaluate and compare the predictive performance of models. In the analyses that used y_adj_ as a pseudo-phenotype, the ratio of the accuracy to the square root of the heritability of the trait [22,23] was computed as a measure of relative accuracy (i.e., the realized accuracy as a fraction of the maximum theoretical accuracy). When the EBV were used as the pseudo-phenotypes, the correlation between EBV and DGV is a non-biased estimate of accuracy [21,24,25].

## 3. Results and Discussion

### 3.1. Descriptive Statistics and Genetic Parameter Estimates

Descriptive statistics for the available phenotypes of OB and IR and their genetic parameter estimates are summarized in Table 2. On average, OB was 27.8 ± 2.4%, indicating that hams lose almost 30% of their initial weight during dry-curing, in agreement with previous reports on Italian dry-cured hams [1]. Values of IR averaged 26.5% and exhibited a lower variability (SD = 1.8%) when compared with OB. The difference between the means of the two traits can be ascribed to a small bias in the infrared predictions, whereas the lower variability of IR compared with OB was expected, IR being a trait predicted with imperfect accuracy. For similar reasons, lower values of both additive genetic and residual variances were expected for IR than for OB. Although the additive genetic variance of IR was 35% lower than that estimated for OB, the heritability (h^2^) for IR was higher (h^2^ = 0.39 for IR, h^2^ = 0.31 for OB) as a consequence of a low residual variance in IR relative to OB (47% of the residual variance of OB). These results are consistent with previous estimates reported for the same pig population [3] and are not surprising, as the heritability of infrared predictions has been reported to be either lower or higher than that of the corresponding measured trait [8,26]. 

### 3.2. Accuracy of Genomic Predictions Assessed in Random Cross-Validation

The accuracies of the genomic predictions obtained for the investigated traits are summarized in Table 3. The five Bayesian models used in this study exhibited similar predictive accuracies. The consistency of results across models, which assumed different prior probability densities for the allele substitution effects [16], may indicate the absence of genomic regions with large effects on the investigated traits or may depend on the size of the dataset used. According to [27], different models tend to show similar predictive ability when the phenotypic variation in the traits is affected by many loci of small effect. This hypothesis is supported by results of genome-wide association studies on ham weight loss after 7 d of salting, which detected a genomic region with the largest effect explaining less than 4% of the genetic variance of the trait [28]. In addition, other studies detected no significant association between ham weight loss after 7 d of salting and SNP genotypes [29]. Regardless of the assumed prior density for the allele substitution effects, in studies using real data the difference in the prediction performance across models is generally small and smaller than that observed in simulation studies [30,31]. In simulation studies (e.g., [27]), where the size of the dataset is not a limiting factor, mixture models performing variable selection (e.g., Bayes B and Bayes C) show better predictive performance than other models. However, when the number of data records is small relative to the number of regression parameters to be estimated, insufficient information from the data restricts the Bayesian learning process. Even though different Bayesian models may lead to quite different estimates of individual allele substitution effects, predictive abilities across models are often very similar when assessed in cross-validation procedures [32]. 

While EBV of CB for OB and IR were predicted with comparable accuracy (scenarios 2 and 5), the ability of the models to predict the CB pre-corrected phenotypes was higher for IR than for OB (scenarios 4 and 1, respectively). Regardless of the model used, prediction accuracies of CB y_adj_ for IR were 65% greater than for OB and were 25% less variable. Such across-trait variation in prediction accuracy might be explained by the combined effect of differences in trait heritability, size of the training set (765 vs. 512 individuals for IR and OB, respectively), and precision of the estimated effects used in the pre-correction of phenotypes resulting from different data availability (9672 vs. 1888 records for IR and OB, respectively).

The investigated genomic models provide a prediction of the additive genetic component of the phenotype, which accounts for 31 and 39% (i.e., the heritability) of the phenotypic variation in OB and IR, respectively. If the true values of the additive genetic component were known and used in the prediction of the phenotype, the accuracy would be maximum and equal to the square root of the trait heritability (i.e., 0.56 and 0.62 for OB and IR, respectively). Then, by assuming that the estimated heritabilities are precise, the prediction accuracies of CB y_adj_ for OB and IR were 31 and 44%, respectively, of the maximum theoretical accuracy (scenarios 1 and 4), indicating that allelic content at the SNPs explained a higher proportion of variation in IR than in OB.

While being just satisfactory when y_adj_ was predicted, accuracies were much higher (0.71 and 0.69 for OB and IR, respectively) when genomic models predicted EBV (scenarios 2 and 5). In agreement with our results, several studies have reported that the choice of the pseudo-phenotype may affect accuracies of genomic predictions to a greater extent than the selection of the modeling approach [32], as the ratio of genetic signal to noise may differ across different pseudo-phenotypes [33]. 

Traits already targeted by genomic selection strategies in pigs were summarized by [12] and include production traits (i.e., loin depth, backfat thickness, carcass weight, average daily gain), functional aspects (i.e., leg score, health traits), meat quality (i.e., pH, marbling, intramuscular fat) and maternal traits (i.e., total number of born, stillborn, pre-weaning mortality, piglet survival). To our knowledge, the performance in prediction of genomic models for WL has never been investigated before and accuracies presented in this study are comparable with those reported for other traits already targeted by genomic selection programs [12], indicating that genomic selection for WL might be successfully implemented. 

The availability of IR offers the opportunity of overcoming difficulties arising from phenotyping for OB which is mandatory when the prediction of genetic merit is provided by pedigree-based methods. Even in a genomic selection framework, phenotyping is essential for the development of prediction equations and for their required periodical update. Hence, assessing the relationship between genomic predictions for IR and pseudo-phenotypes of OB (scenarios 7, 8, and 9) was an important objective of this study. For models trained on y_adj_ for IR, the correlation between the prediction and y_adj_ of OB (scenario 7) was slightly greater than that for models trained on y_adj_ for OB (scenario 1). Conversely, the use of the EBV for IR in model training (scenario 8), compared to the use of the EBV for OB (scenario 2), resulted in a 25% lower correlation between the DGV and the EBV for OB. Such inconsistency may be attributed to the difference in the predictive accuracy of IR and OB arising from the training on different pseudo-phenotypes. While the genomic predictions of EBV were of similar accuracy for IR and OB, predictions of y_adj_ were much more accurate (+65%) for IR than for OB.

Accuracies in the prediction of the EBV of the purebred animals were investigated in scenarios 3, 6, and 9. In the breeding program of the investigated boar line, the genetic evaluation of purebred breeding candidates relies on the phenotypic information provided by their paternal CB half-sibs generated by the C21 nucleus boars. Under such circumstances, where all members of a sire purebred family acquire identical EBV, assessing the ability of genomic models to predict the EBV of the purebred breeding candidates is challenging because of the moderate accuracy of the EBV that must be predicted. Hence, validation of the genomic models in scenarios 3, 6, and 9 focused on the PB (i.e., the C21 nucleus boars) for which the accuracy of the EBV is high (on average 0.75 for IR and 0.72 for OB). 

In the random cross-validation, prediction accuracies for the EBV of PB were much higher and less variable than those for the EBV of CB and greater than 0.90 when models were trained and validated within trait (scenarios 3 and 6). Such difference in the predictive accuracies of the EBV for CB and PB can be ascribed to the characteristics of the cross-validation procedure which favored the predictive ability of the models for the EBV of PB. Indeed, at each round of the cross-validation, approximately 80% of the progeny of each PB was in the training set used to obtain the model solutions leading to the prediction of the EBV of the PB. For the CB, the data of the animals in the validation set were omitted from the training set, weakening to a greater extent than for the PB the link between the information used for model building and the EBV to be predicted. As expected, the ability of models trained on the EBV for IR to predict the EBV for OB of the PB (scenario 9, r = 0.675) was lower than that exhibited by models trained on the EBV for OB (r = 0.937). This loss in accuracy (−28%) was equal to that detected for the EBV of CB. 

The accuracy estimates obtained in the random cross-validation procedures are optimistic, as individuals closely related (e.g., full and half-sibs) to the animals requiring a prediction may end up in the training set which is unrealistic in a real genomic selection scenario. Family-based linkage disequilibrium enhances the prediction ability of genomic models when compared with circumstances where only population-wide linkage disequilibrium supports the predictive strength of models.

### 3.3. Model Accuracy in a Leave-One-Family-Out Validation Scheme

Results obtained in the LOFO training-validation scheme are reported in Table 4. For OB, the correlation between DGV and y_adj_ of CB (scenario 1) was 0.23, indicating a moderate ability of the model to predict the phenotypic variation in OB. This estimate was higher than the one (r = 0.17) obtained for the same scenario in the random cross-validation. In the random cross-validation procedure, the training set for OB consisted on average of 512 animals, as 20% of the data were removed at each round and used for model validation. The training sets in the LOFO scheme were of greater size than in the random cross-validation as animals of only one sire family (on average 35 pigs) were discarded from the training set. This explains the increase in the predictive ability of the models detected in the LOFO procedure. 

The correlation between DGV and EBV of CB for OB (scenario 2) was 0.45. This correlation is an estimate of model predictive accuracy under more restrictive training-validation conditions than those of random cross-validation. The correlation was 0.71 when the predictive accuracy was assessed with random cross-validation, where animals of each sire family were randomly assigned to the training and validation set. In the LOFO, data of full-sibs and paternal half-sibs of the animals allocated to the validation set did not contribute to the development of the genomic prediction equation. The breakage of the family-based linkage disequilibrium caused by the LOFO explains the decrease in accuracy detected in such a validation scheme. This result indicates also that the increase in the size of the training set occurring in the LOFO was not able to compensate for the loss in EBV prediction accuracy due to the absence of close relatives in the training set.

In the population investigated in this study, purebred candidates are selected based on the performance of their CB relatives generated in the sib-testing program, thus no phenotypes for ham quality traits are available for purebred pigs. Under these circumstances, the genetic merit of purebred breeding animals has to be predicted using a training population of CB only. The correlation between DGV and EBV of PB for OB in scenario 3 corresponds to the accuracy of models predicting the genetic merit of purebred animals from a reference population of CB exhibiting no close additive genetic relationships with the PB, thus representing the most realistic scenario. For OB, the correlation between DGV and EBV of PB was approximately 0.38, indicating that the model can predict the genetic merit of the PB sires with a small loss in accuracy when compared to that achieved for the CB (0.45). 

When the model was trained and tested on the IR (scenarios 4–6), accuracies detected in the LOFO scheme were consistent with those obtained for a model trained and tested on OB (scenarios 1–3). As expected, accuracies obtained in scenarios 7–9 were lower than those obtained in scenarios 1–3 and 4–6 (Table 4). The greatest decrease in accuracy was for y_adj_ (−32%). The correlation between DGV for IR and EBV of PB for OB (scenario 9) approximates the accuracy of a genomic prediction model trained on IR phenotypes to predict the EBV of PB for OB. The estimated correlation was 0.32, just 15% lower than the one achieved when the model was trained on EBV for OB (r = 0.38, scenario 3).

For traits in the same range of heritability (from 0.3 to 0.4, e.g., average daily gain, backfat thickness, lean %, feed conversion rate, ultrasound muscle depth), when GBLUP or Bayesian regression models were trained on a reference population of size comparable to or greater than that of our population and validated on animals born after a cut-off date, accuracies of prediction of phenotype (adjusted for fixed effects) ranged from 0.1 to 0.4 [34,35,36], in line with our results. In those studies, as the training and validation set were defined based on a cut-off date of birth of the genotyped animals, pigs in the validation set had close relatives in the training set [37], representing a more favorable condition than the one investigated in the current study. To the best of our knowledge, no studies investigated the accuracy of models trained on one trait with the aim to predict the pseudo-phenotype of a different trait.

The IR records for each slaughter batch were generated through infrared prediction models trained on phenotypes from other batches. Therefore, the accuracy of such models has been deliberately reduced in order to mimic the conditions under which models are applied in practice. Compared to the random allocation of samples to the training set reported by [3], this procedure has weakened the correlation between IR and OB, which dropped from 0.79 [3] to 0.65 in the current study. However, despite the moderate phenotypic relationship between IR and OB, the additive genetic correlation between the two traits was large and positive. In particular, it was 0.85 in the current study, not significantly different from the estimate (0.88) obtained by [3]. Such favourable genetic correlation confirms the potential of IR as an indicator trait for OB in pig breeding programs aiming at decreasing WL and can explain the results obtained in scenario 9. Despite the limitations to the accuracy of the infrared prediction models imposed by our procedure and the breakage of the family-based linkage disequilibrium generated by the LOFO procedure, the results of this study confirm that genomic selection for WL would benefit from the use of IR phenotypes in the development of genomic prediction models.

Genomic selection in animal breeding programs is expected to exert a beneficial impact on two key aspects: the length of the generation interval and the accuracy of selection. In pigs, improvements of the rate of genetic progress through a reduction of the generation interval are usually limited, given the short generation intervals (<2 years) that the traditional breeding programs can reach [12], but traits such as WL, for which OB phenotypes are collected 13–14 months after slaughter, would benefit considerably from the adoption of genomic selection, as it would reduce the generation interval by approximately 50%. In addition, in pigs, the accuracy of the DGV tends to decline rapidly due to rapid generation turnover, and periodical phenotype collections, especially in animals related to selection candidates, are likely to be needed to update genomic prediction models [14,15,32]. Routine phenotyping for OB is currently unfeasible and periodical phenotyping is expensive and requires the completion of the seasoning period, with delays in the updating of genomic prediction models. In addition, when the target is the prediction of PB genetic merit for CB animals, as in our case, an increased number of training animals with phenotypes is needed. Under these circumstances, the use of IR in place of OB in model training results in a reduction of the phenotyping costs, favoring the construction of larger reference populations, greater flexibility in the collection of phenotypes, and faster updates of the prediction models, with only a minimal impact on model accuracy compared to the use of OB phenotypes. 

## 4. Conclusions

The main aims of this study were to investigate the accuracy of genomic predictions of WL phenotypes or pseudo-phenotypes achieved using IR or OB in the training of genomic models and to assess the loss in accuracy when the prediction of OB was obtained from models trained on IR.

Accuracies of genomic predictions of OB and IR were moderate to high in a random cross-validation setting and decreased by a similar extent in the leave-one-family-out scenario, indicating that genomic predictions of IR or OB may be exploited in selective breeding aiming at reducing WL in dry-curing. However, despite limitations imposed to the accuracy of the infrared predictions, the moderate size of the reference population used in model training, and the penalizing conditions due to the leave-one-family-out procedure, the accuracy of selection guaranteed by genomic-predicted EBV for IR exhibited just a minimal loss when compared to that observed for models trained on actual measures of WL. The use of IR in place of OB facilitates periodical model retraining, enables higher flexibility in phenotype collection at reduced costs, while maintaining accuracies comparable to those achieved with measures of WL. In the view of implementing IR in genomic selection for WL, periodical model retraining, based on new samples, must be envisaged not only for the genomic prediction tools but also for infrared prediction models. Future studies should elucidate how frequently infrared prediction models must be retrained to keep the predictive ability of genomic models based on IR at the optimal level. Implementation of IR would have a limited impact on the running costs of either an existing genomic selection program or an established traditional breeding program focused on ham quality while making the genetic improvement of WL feasible.

## Figures and Tables

**Table 1:** Training-validation scenarios used for the genomic regression models.

Scenario	Training ^1^				Validation ^2^		
	Trait	Pseudo-Phenotype	Pigs		Trait	Pseudo-Phenotype	Pigs
1	OB	y_adj_	CB		OB	y_adj_	CB
2	OB	EBV	CB		OB	EBV	CB
3	OB	EBV	CB		OB	EBV	PB
4	IR	y_adj_	CB		IR	y_adj_	CB
5	IR	EBV	CB		IR	EBV	CB
6	IR	EBV	CB		IR	EBV	PB
7	IR	y_adj_	CB		OB	y_adj_	CB
8	IR	EBV	CB		OB	EBV	CB
9	IR	EBV	CB		OB	EBV	PB

^1^ Models for observed (OB) and infrared-predicted (IR) ham weight loss were trained either on the pre-corrected phenotype (y_adj_) or on the estimated breeding value (EBV) of crossbred pigs (CB); ^2^ The prediction equation obtained from the analysis of the training set was applied to genotypes of CB or of their purebred sires (PB) to predict the pseudo-phenotype. The accuracy in predicting y_adj_ (for CB) or EBV (for CB and PB) was assessed as the correlation between the predicted and the observed pseudo-phenotype in the validation set using either a 5-fold cross-validation or a leave-one-family-out validation procedure.

**Table 2:** Descriptive statistics, additive genetic variance ($\sigma_{a}^{2}$ ), residual variance ($\sigma_{e}^{2}$ ), and heritability (h^2^) for observed ham weight loss (OB) and its infrared spectroscopy prediction (IR).

Trait	n	Mean	SD	Min	Max	$\sigma_{a}^{2}$	$\sigma_{e}^{2}$	**h^2^ ± SE**
OB, %	1888	27.8	2.4	20.1	38.2	1.468	3.237	0.312 ± 0.053
IR, %	9672	26.5	1.8	19.5	34.1	0.961	1.513	0.388 ± 0.031

**Table 3:** Correlations (±SE) between direct genomic values (DGV) and pre-corrected phenotypes (y_adj_) or estimated breeding values (EBV) for observed (OB) and infrared-predicted (IR) ham weight loss estimated in the 5-fold cross-validation for different models.

Type of DGV ^2^	Correlation with	Model ^1^				
		BRR	BA	BB	BC	BL
of CB for OB	y_adj_ of CB for OB	0.170 ± 0.035	0.172 ± 0.035	0.171 ± 0.038	0.172 ± 0.036	0.173 ± 0.034
of CB for OB	EBV of CB for OB	0.708 ± 0.023	0.709 ± 0.023	0.708 ± 0.024	0.707 ± 0.023	0.709 ± 0.023
of PB for OB	EBV of PB for OB	0.937 ± 0.004	0.937 ± 0.004	0.937 ± 0.004	0.937 ± 0.004	0.938 ± 0.004
of CB for IR	y_adj_ of CB for IR	0.277 ± 0.028	0.276 ± 0.028	0.276 ± 0.030	0.276 ± 0.028	0.276 ± 0.027
of CB for IR	EBV of CB for IR	0.689 ± 0.008	0.689 ± 0.008	0.689 ± 0.008	0.689 ± 0.008	0.689 ± 0.008
of PB for IR	EBV of PB for IR	0.914 ± 0.003	0.913 ± 0.003	0.913 ± 0.003	0.912 ± 0.004	0.913 ± 0.003
of CB for IR	y_adj_ of CB for OB	0.210 ± 0.020	0.210 ± 0.020	0.207 ± 0.020	0.208 ± 0.020	0.210 ± 0.021
of CB for IR	EBV of CB for OB	0.520 ± 0.017	0.520 ± 0.016	0.519 ± 0.017	0.519 ± 0.017	0.521 ± 0.016
of PB for IR	EBV of PB for OB	0.675 ± 0.014	0.675 ± 0.014	0.673 ± 0.013	0.671 ± 0.014	0.677 ± 0.014

^1^ BRR: Bayesian Ridge Regression; BA: Bayes A; BB: Bayes B; BC: Bayes C; BL: Bayesian LASSO; ^2^ CB: crossbred pigs; PB: purebred sires of crossbred pigs.

**Table 4:** Correlations (r) between direct genomic values (DGV) and pre-corrected phenotypes (y_adj_) or estimated breeding values (EBV) for observed (OB) and infrared-predicted (IR) ham weight loss estimated in the leave-one-family-out validation ^1,2^.

Type of DGV ^3^	Correlation with	n	r	95% CI	*p*-Value ^4^
of CB for OB	y_adj_ of CB for OB	640	0.229	0.154–0.301	<0.001
of CB for OB	EBV of CB for OB	735	0.447	0.384–0.505	<0.001
of PB for OB	EBV of PB for OB	57	0.383	0.136–0.585	<0.01
of CB for IR	y_adj_ of CB for IR	956	0.224	0.163–0.284	<0.001
of CB for IR	EBV of CB for IR	995	0.437	0.385–0.486	<0.001
of PB for IR	EBV of PB for IR	104	0.396	0.220–0.547	<0.001
of CB for IR	y_adj_ of CB for OB	606	0.156	0.077–0.233	<0.001
of CB for IR	EBV of CB for OB	995	0.351	0.295–0.405	<0.001
of PB for IR	EBV of PB for OB	104	0.318	0.134–0.481	<0.001

^1^ DGV were obtained from solutions for allele substitution effects estimated with a Bayesian Ridge Regression genomic model; ^2^ n: number of animals used to compute the correlation; 95% CI: 95% confidence interval of the correlation; ^3^ CB: crossbred pigs; PB: purebred sires of crossbred pigs; ^4^ *p*-value for the null hypothesis H_0_: r = 0.

## Data Availability

Restrictions apply to the availability of these data. Data was obtained from Gorzagri (Fonzaso, Italy) and are available from the authors with the permission of Gorzagri.
